# Biomarkers of Immune Injury in Cardiometabolic Disorders

**DOI:** 10.3390/ijms27219913

**Published:** 2026-11-05

**Authors:** Letao Zhou, Yifei Li

**Affiliations:** Key Laboratory of Birth Defects and Related Diseases of Women and Children of MOE, Department of Pediatrics, West China Second University Hospital, Sichuan University, Chengdu 610041, China; zzlt_zhou@163.com

**Keywords:** cardiometabolic disorders, immune injury, biomarkers, immunometabolism, inflammation, risk stratification

## Abstract

Cardiometabolic disorders are accompanied by persistent immune activation that links metabolic stress to vascular and organ injury. Conventional inflammatory measures capture part of this process, but they provide limited information about tissue origin, biological mechanism and disease trajectory. This narrative review examines emerging biomarkers across genetic and epigenetic regulation, metabolic and oxidative stress, gut microbial exposure, innate immune activation, adaptive immune remodeling and circulating inflammatory responses. It integrates mechanistic studies with clinical observations to consider how these signals may inform the assessment of cardiometabolic immune injury. Particular attention is given to the distinction between inherited susceptibility and acquired responses, circulating associations and tissue mechanisms, and potentially injurious versus compensatory immune activity. The evidence supports substantial biological connectivity among these domains, while the strength of clinical validation varies widely. Most candidate markers and proposed combinations require standardized measurement, longitudinal assessment and external validation before biomarker-guided prevention can be justified. We propose a mechanism-informed framework for organizing candidate immune-metabolic phenotypes, recognizing reciprocal interactions rather than a universal temporal sequence. Future studies should determine whether these biomarkers improve prediction and clinical decisions beyond established risk factors and whether changes in biomarker profiles correspond to meaningful treatment benefits.

## 1. Introduction

Cardiometabolic disorders encompass a tightly connected spectrum of obesity, insulin resistance, type 2 diabetes, dyslipidemia, hypertension, metabolic dysfunction-associated steatotic liver disease, chronic kidney disease and cardiovascular disease. Their clinical importance lies not only in the clustering of conventional risk factors but also in progressive injury across the vasculature, myocardium, kidney, liver and skeletal-muscle system. In a multi-biomarker study of incident atherosclerotic cardiovascular disease, Chen and colleagues linked metabolic, inflammatory and lipoprotein(a)-related risk profiling to subsequent vascular events, while the Kailuan cohort showed that metabolic syndrome in the context of higher hsCRP was associated with increased heart-failure risk [1,2]. Consistent with this continuum, cumulative hsCRP has been examined in relation to all-cause mortality among middle-aged and older adults, and a national study of the spectrum of cardiometabolic disorders evaluated inflammatory pathways in the association between environmental exposures and mortality [3,4]. These observations indicate that cardiometabolic disease is a multisystem disorder in which inflammatory burden accompanies, and may accelerate, adverse clinical trajectories. In Negrito adults, the IDF and JIS definitions of metabolic syndrome showed high agreement, whereas agreement with cardiovascular risk scores was limited. Another study linked metabolic syndrome and sarcopenia through genetic and transcriptomic features [5,6]. Associations among gait speed, CRP and metabolic syndrome in older Chinese adults provide additional clinical context for skeletal-muscle function [7].

In this review, immune injury refers to tissue damage or impaired organ function arising in part from dysregulated immune responses. Immune activation and inflammation describe responses that may be protective or reversible; they do not, by themselves, demonstrate injury. We interpret a biomarker as an indicator of immune injury when its immune relevance is supported by experimental or tissue evidence and it is associated with a measurable structural or functional abnormality, such as histological fibrosis, impaired vascular function or a sustained decline in glomerular filtration. These abnormalities indicate injury but do not identify its cause. Longitudinal associations strengthen the link between a biomarker and injury progression, whereas a causal role requires additional mechanistic evidence. Markers linked only to metabolic traits or circulating inflammation are therefore discussed as correlates or candidates. This is a working definition for the review, rather than a validated diagnostic criterion.

Immune injury is increasingly recognized as a feature of cardiometabolic dysfunction rather than an incidental consequence of established organ disease. Nutrient excess, lipotoxicity, hyperglycemia, oxidative stress and altered microbial exposure affect adipocytes, hepatocytes, renal tubular epithelial cells, vascular endothelial and smooth-muscle cells, and tissue-resident macrophages. These cellular perturbations recruit and reprogram circulating monocytes, neutrophils and lymphocytes, creating persistent low-grade inflammatory activation. In cardiovascular–kidney–metabolic (CKM) syndrome, the CRP–triglyceride–glucose index has been evaluated across CKM stages as an integrated inflammation–insulin-resistance measure; similarly, PTX3 has been evaluated for differentiating CKM stages [8,9]. At the cellular level, NLRP3-containing extracellular vesicles from patients with metabolic syndrome have been implicated in atherosclerosis development, supporting the concept that immune injury is propagated between metabolically stressed tissues and the vascular wall rather than restricted to a single organ [10]. Studies of the TyG index, inflammatory markers and adipokines in metabolic syndrome provide cross-sectional associations rather than evidence of the temporal sequence of immune injury [11].

This immune remodeling is clinically consequential. Inflammatory activation can amplify endothelial dysfunction, plaque instability, myocardial remodeling, hepatic fibrogenesis and loss of renal function through inflammasome activity, cytokine release, complement activation and protease-mediated matrix injury. Prospective analyses have associated systemic hematologic inflammation measures, including SII and SIRI, with all-cause and cardiovascular mortality across the spectrum of cardiometabolic disorders, whereas MHR has been linked to inflammatory disease and disease-specific mortality. These associations link immune-related biomarkers to adverse outcomes but do not by themselves establish that the measured signals cause organ injury [12,13,14]. Early recognition of immune injury may improve risk stratification before irreversible organ disease. Conventional glucose and lipid measures incompletely reveal trained innate activation, adaptive dysregulation, gut-barrier failure or tissue remodeling. The candidate biomarkers discussed here include genetic and epigenetic measures, metabolic and oxidative-stress markers, microbiome- and barrier-related markers, innate immune mediators, adaptive immune-cell and antibody profiles, and circulating inflammatory proteins and blood-cell indices. Their clinical value depends on reproducibility, tissue relevance and predictive gain beyond conventional assessment [10,15,16].

The order of the following sections is used to organize the evidence by biological process and measurement type; it does not imply a universal sequence of disease development. The framework considers reciprocal interactions among these processes, whose temporal relationships may differ across tissues and disease stages.

### 1.1. Literature Search and Selection

This narrative review brings together evidence on immune dysregulation and related biomarkers in cardiometabolic disorders. The review covers literature published through September 2026, with emphasis on relevant studies from the preceding 10 years; earlier foundational studies are retained where they clarify key immune mechanisms. The literature covers genetic and epigenetic regulation, metabolism and oxidative stress, the gut microbiota and barrier function, innate and adaptive immunity, and circulating inflammatory mediators and blood-cell-derived indices. Studies are considered in relation to a defined question about biomarker biology, measurement or clinical relevance, rather than an association with inflammation alone. SANRA provides a framework for assessing the clarity of the review’s aims, literature search, referencing, scientific reasoning and presentation of relevant outcome data [17].

### 1.2. Framework for Interpreting Biomarker Evidence

Different study designs answer different questions in this review. Experimental studies inform mechanisms, population studies assess associations with phenotypes and subsequent outcomes, and intervention studies examine treatment effects and biomarker responses. Methodological studies address measurement reliability. For prognostic and clinical interpretation, greater weight is given to prospective human studies with relevant outcomes than to cross-sectional associations or experimental findings. Evidence from selected clinical populations is interpreted within its disease and treatment context, with broader applicability considered separately. Cross-sectional discrimination does not establish prognosis or causality, and a treatment-associated change does not establish differential treatment benefit. Longitudinal associations should be distinguished from incremental prediction and external validation; clinical utility concerns whether using a test improves decisions and outcomes. Table 1 summarizes the evidence categories and their limits.

The biological meaning of a biomarker depends on both the sampled compartment and the evidence for its origin. Circulating proteins and metabolites describe the blood compartment, whereas cellular markers characterize the abundance or state of the cells examined. EV-associated markers describe vesicle composition or cargo; assigning them to a particular cell or tissue requires evidence of origin. Tissue measurements provide local evidence, but a corresponding blood measurement does not necessarily identify the same process. Microbiome-related markers include microbial products and metabolites formed through host–microbial metabolism, such as TMAO. These categories overlap: a microbial metabolite or an EV-associated signal may also be measured in blood. Experimental cellular models help explain how a signal may arise or act but do not by themselves establish its source in patients. Keeping these distinctions explicit allows circulating measurements to be linked to organ phenotypes without assuming that they directly measure the underlying tissue lesion.

For circulating molecular markers, concentrations reflect release and clearance as well as immune activity. Depending on the analyte, altered clearance, release from injured tissue, systemic acute-phase responses and medication exposure may therefore offer alternative explanations for an association. Disease-related changes can also alter biomarker levels, so the direction of an observed association should not be assumed; tissue source and causality require separate evidence.

## 2. Genetic and Epigenetic Reprogramming

Genetic and epigenetic signals are considered here as regulators and markers of susceptibility to cardiometabolic immune injury. Germline variants establish susceptibility in monocytes, macrophages, endothelial cells, adipocytes, hepatocytes and pancreatic islets; methylation, histone-associated transcriptional control and non-coding RNAs then record the effects of nutrient excess, glucolipotoxicity and oxidative stress [35]. These processes can alter the activation threshold of innate sensors and antigen-presenting cells, but their relationship to immune remodeling varies with the tissue and disease context. Their value as biomarkers lies in detecting durable regulatory potential—rather than transient cytokine output—and in identifying people likely to generate exaggerated immune injury when exposed to metabolic stress. Blood is practical for such assays, but tissue specificity remains crucial: leukocyte methylation largely indexes circulating immune programming, whereas liver-, adipose- or endothelial-derived extracellular RNA may better reflect organ-local cardiometabolic injury [15,36,37].

### 2.1. DNA Methylation and Epigenetic Age as Early Indicators of Inflammatory Metabolic Programming

Methylation patterns and epigenetic clocks may capture persistent regulatory changes associated with metabolic exposures [15,35,38]. Associations of genetic risk scores with inflammatory and oxidative-stress markers in people with diabetes, and a genetic study of CRP in Indian adolescents, indicate that population and age differences require examination [39,40].

Methylation at immune and acute-phase loci is relevant before overt diabetes or cardiovascular disease. In 40 participants, Carraro et al. associated global, IL6, SERPINE1 and CRP methylation with adiposity, insulin resistance and diet; CRP methylation discriminated insulin resistance better than circulating CRP in this cross-sectional sample [38]. This supports a regulatory readout beyond acute-phase concentrations. Nutrient excess, oxidative stress and cytokines may alter methyltransferase activity and chromatin accessibility, linking metabolic exposure to inflammatory inducibility [35]. Epigenetic age acceleration provides a systemic indicator of accumulated injury. In 179 veterans followed for two years, greater baseline Hannum DNA-methylation age acceleration predicted worsening metabolic-syndrome severity, including lipids and abdominal obesity; baseline inflammatory and metabolic measures did not predict later epigenetic aging [15]. The absence of corresponding Horvath-clock associations underscores phenotype-specific clock selection. Clinical translation requires standardized preprocessing, cell-composition adjustment and prospective validation.

### 2.2. Inherited Inflammatory Variation and Non-Coding RNAs

Genome-wide association approaches can identify inherited determinants of inflammatory tone that may modify susceptibility to adipose inflammation, insulin resistance and atherosclerosis. In an admixed Brazilian population, Leite et al. identified 12 suggestively associated single-nucleotide polymorphisms for eight inflammatory biomarkers, including the missense OR6K6 variant rs16841009 associated with monocyte chemoattractant protein-1 (MCP-1) [36]. MCP-1/CCL2 recruits CCR2-positive monocytes into adipose tissue and the vascular wall; a genetic tendency toward higher MCP-1 could therefore favor macrophage infiltration, adipocyte dysfunction and plaque inflammation. The clinical role of individual variants remains limited because effect sizes are generally modest and ancestry-specific linkage patterns can influence interpretation. However, pathway-based genetic scores that incorporate chemokine, inflammasome, lipid-metabolism and complement loci may eventually distinguish life-long inflammatory susceptibility from inflammation primarily driven by current obesity, diet or renal dysfunction. A study of TNF-α rs1800629 in middle-aged women examined its relationship with inflammatory biomarkers but did not confirm an overall direct association [41].

Non-coding RNAs provide a dynamic bridge between inherited susceptibility and environmental programming. In fructose-fed rats and fructose-exposed hepatocyte models, reduced miR-200a relieved repression of thioredoxin-interacting protein (TXNIP), promoted NLRP3 inflammasome activation and aggravated hepatic inflammation; curcumin and allopurinol increased miR-200a and attenuated the TXNIP/NLRP3 axis [18]. This mechanism links fructose-associated redox stress to inflammasome activation and suggests that miR-200a may report a molecular state of oxidative hepatic injury. In children with obesity, lower PBMC miR-155 expression was associated with obesity, higher hsCRP and greater fat mass, whereas miR-122 and miR-193a were higher in participants with metabolic syndrome, insulin resistance or high fat mass and correlated with fasting insulin, HOMA-IR and triglycerides [42]. These findings identify PBMC microRNA expression patterns associated with metabolic and inflammatory traits but do not establish immune-cell reprogramming or predict subsequent clinical events. In older adults, multiple circulating microRNAs including miR-15a, miR-16, miR-223, miR-363 and miR-532 were inversely associated with leptin and/or a low-grade systemic inflammation score, while miR-21 was positively associated with inflammation [37]. These associations link circulating microRNA profiles to leptin and low-grade inflammation, without establishing leptin resistance or a transition between immune states. Long non-coding RNAs provide related information: in metabolic syndrome, adiponectin antisense RNA and MALAT1 were positively associated with inflammatory measures, while MALAT1 was inversely associated with serum 25-hydroxyvitamin D [43]. These associations connect non-coding RNA expression with inflammatory and metabolic measures but do not establish the cellular source or the underlying pathway. Post-translational glycomic signatures should be considered in this regulatory domain because immunoglobulin-G N-glycosylation influences Fc-receptor binding, complement activation and antibody effector function. In a community-based Chinese Han cohort of 701 participants, IgG N-glycan biosignatures differentiated metabolic syndrome, hypertriglyceridemic waist and abdominal obesity from controls with area-under-the-curve values of 0.632, 0.659 and 0.610, respectively [24]. Although discrimination was modest, the biological rationale is strong: reduced galactosylation or altered fucosylation can favor pro-inflammatory antibody activity.

### 2.3. Translational Use of Regulatory Biomarker Panels

Regulatory biomarkers may explain why similar metabolic phenotypes diverge. Whether regulatory profiles explain these differences requires testing against tissue and clinical outcomes [15,24]. Cell composition is critical because apparent methylation differences may arise from altered proportions of neutrophils, monocytes, T cells or B cells rather than from cell-intrinsic programming [21]. Likewise, microRNA levels can originate from platelets, leukocytes, endothelium, adipose tissue or EVs, so their relationship to disease requires parallel cellular or vesicle characterization. Regulatory biomarkers may clarify intervention response even when CRP changes little. Repeated methylation and microRNA measurements could test whether weight loss, exercise or dietary intervention attenuates immune-memory pathways. Dietary trials and PREDIMED-Plus analyses demonstrate inflammatory-marker changes, without establishing parallel epigenetic changes [44,45]. A short pomegranate-extract trial also assessed inflammation and blood pressure in older adults [46]. Regulatory multi-omics could thus complement clinical monitoring and trial enrichment.

## 3. Metabolic and Oxidative-Stress Signatures

Metabolic and oxidative-stress signals reflect disturbances in substrate handling and redox balance in cardiometabolic disease. Adipocyte hypertrophy, impaired skeletal-muscle substrate oxidation, hepatic lipogenesis, renal clearance defects and mitochondrial overload change the extracellular nutrient and redox environment and interact with immune responses. BCAAs, urate, oxidized lipoproteins, ceramides, glycation products, GlycA-related acute-phase glycosylation and redox products therefore report the biochemical terrain that trains macrophages, injures endothelial cells and sensitizes inflammasome pathways. These measurable signals can be related to metabolic and organ phenotypes [47,48]. A coronary CT study in premenopausal women with metabolic syndrome linked composite metabolic indices and oxidative stress to subclinical coronary atherosclerosis, but its cross-sectional associations do not establish the sequence of injury [49].

### 3.1. Inflammatory Metabolite Signatures and Amino-Acid Dysregulation

Metabolic biomarkers are central to the immune injury framework because metabolites can directly activate inflammatory pathways, impair mitochondrial function, alter endothelial behavior and influence immune-cell differentiation. Glycoprotein acetyls, serum urate, branched-chain amino acids (BCAAs), advanced glycation end products, adipokines and integrated metabolomic signatures provide information beyond fasting glucose or triglycerides. They identify inflammation-related glycosylation, purine/redox stress, impaired nutrient oxidation, carbonyl stress and pathway remodeling that may precede overt organ damage [27,47,50]. GlycA is a composite NMR signal generated by N-acetyl methyl groups on several acute-phase glycoproteins, particularly α1-acid glycoprotein, haptoglobin, α1-antitrypsin and transferrin. Because it integrates multiple proteins, GlycA may be less susceptible than a single cytokine to transient biological fluctuations. In the METSIM cohort, GlycA, IL-1 receptor antagonist (IL-1RA) and hsCRP were assessed against insulin secretion, insulin sensitivity, incident type 2 diabetes, hypertension, cardiovascular events and mortality; a principal-component score combining these markers was associated with subsequent disease outcomes [27]. The observation indicates that glycoprotein acetylation, IL-1-pathway activity and the hepatic acute-phase response encode partially distinct dimensions of low-grade inflammation. The complementary associations of GlycA, IL-1RA and hsCRP with subsequent outcomes provide a rationale for combining these markers to refine cardiometabolic risk assessment. Whether this combination improves individual risk estimates beyond established clinical factors remains to be determined. BCAAs are associated with insulin resistance and may signal reduced mitochondrial BCAA oxidation, defective adipose and muscle catabolism and activation of nutrient-sensing pathways. In 19,472 women without baseline diabetes, cardiovascular disease or cancer, the highest versus lowest BCAA quartile was associated with higher hsCRP (1.96 versus 1.43 mg/L), fibrinogen (367 versus 362 mg/dL), soluble ICAM-1 (361 versus 353 ng/mL) and GlycA (407 versus 371 μmol/L), as well as lower HDL cholesterol and higher triglycerides, LDL cholesterol and lipoprotein insulin-resistance score [47]. These associations persisted after adjustment for established cardiovascular risk factors. BCAA profiling may consequently identify a multi-pathway phenotype of impaired nutrient oxidation, endothelial activation, dyslipidemia and low-grade inflammation, but prospective work must determine whether BCAA trajectories predict diabetes, steatotic liver disease or vascular events beyond glycemia and adiposity.

### 3.2. Urate, Glycation and Oxidative Stress as Inflammatory Metabolic Danger Signals

Serum urate exemplifies a metabolic product whose prognostic meaning depends on concurrent inflammation. In 2731 non-diabetic adults, urate was positively associated with hsCRP, fibrinogen, ferritin, complement C3 and erythrocyte sedimentation rate after adjustment for age, sex, adiposity and diuretic or antihypertensive use [50]. In HepG2 cells, urate dose-dependently increased CRP, fibrinogen, ferritin and C3 expression through IκB kinase/IκBα/NF-κB signaling; inhibition of urate transport or reactive oxygen species with N-acetylcysteine attenuated the effect. Hyperuricemia may therefore indicate both impaired renal/metabolic handling and a capacity to amplify hepatic innate-inflammatory output. A large prospective coronary-artery-disease study illustrates the interaction between urate and systemic inflammation. Among 16,598 participants, increasing serum-urate quintiles were associated with higher two-year major adverse cardiovascular and cerebrovascular event rates only when hsCRP was at least 2 mg/L [28]. Within this inflammatory stratum, each 1 mg/dL increase in urate was associated with approximately 13% higher adjusted MACCE risk (HR 1.13, 95% CI 1.08–1.18; interaction *p* = 0.002); the association was absent in those with hsCRP below 2 mg/L. Thus, urate alone is insufficiently specific, whereas the urate–CRP combination identifies a phenotype in which purine-related stress and systemic inflammation coexist. This type of effect modification is instructive for biomarker development: a metabolite becomes clinically informative when interpreted in the biological context that determines its downstream inflammatory consequences. Advanced glycation end products (AGEs) and oxidized lipids form another metabolic danger network. AGE engagement of RAGE promotes oxidative stress, NF-κB activation, endothelial dysfunction and inflammatory-gene transcription. Oxidized LDL, malondialdehyde and lipoprotein-associated phospholipase A2 (Lp-PLA2) can report oxidized-lipoprotein burden and vascular inflammatory activity. In a randomized double-blind trial in metabolic syndrome, a red-yeast-rice–olive-extract supplement reduced OxLDL and Lp-PLA2, without significant reductions in malondialdehyde or urinary 8-OHdG [31]. This short trial supports investigating OxLDL and Lp-PLA2 as pharmacodynamic measures, but outcome trials are required before changes in OxLDL, Lp-PLA2 or redox indices can be interpreted as evidence of reduced event risk. A randomized trial in 38 adults with overweight or obesity also compared low and high dietary AGE intake in relation to insulin sensitivity and inflammatory measures [51]. Population associations of vitamin E with inflammatory markers and metabolic syndrome cannot be interpreted directly as effects of supplementation [52].

### 3.3. Lipidomics, Extracellular-Vesicle Cargo and Metabolomic Prediction

Lipidomics offers higher mechanistic resolution than conventional lipids because ceramides, sphingolipids, lysophospholipids and oxidized phospholipids can alter membrane signaling, mitochondrial bioenergetics and immune-cell behavior. Ceramide accumulation can impair insulin signaling and promote macrophage, hepatic and endothelial inflammatory responses. In 57 overweight or obese adults evaluated before and after 29–30 days of Ramadan intermittent fasting, inflammatory markers and routine lipid measures improved alongside declines in sphingosine, sphinganine, sphingosine-1-phosphate, sphinganine-1-phosphate and selected sphingomyelin and dihydrosphingomyelin species [32]. This indicates that individual sphingolipids can act as dynamic markers of dietary remodeling, though the study was not designed to assess hard outcomes. A cross-sectional analysis of serum omega-6 polyunsaturated fatty acids and inflammatory markers suggests that lipid composition is related to inflammatory status but does not justify treating all omega-6 fatty acids as pro-inflammatory exposures [53]. Extracellular-vesicle (EV) lipidomics may further reveal inter-organ transmission of metabolic injury. EVs from adiponectin-deficient mice were enriched in sphingolipids and polyunsaturated phospholipids; they increased macrophage reactive oxygen species and NF-κB/IRF signaling, failed to increase ATP production in skeletal-muscle cells and impaired glucose-stimulated insulin secretion in β cells [48]. EVs from patients with metabolic syndrome showed parallel remodeling, particularly enrichment of PUFA-containing lipids. The adiponectin–EV lipid axis therefore offers a plausible mechanism through which loss of protective adipose signaling distributes pro-oxidative and pro-inflammatory cargo to distant tissues. However, EV isolation, lipoprotein contamination and pre-analytic variation remain major barriers to clinical deployment. Untargeted metabolomics can identify response signatures that exceed the information contained in a single analyte. In 50 Chinese men with metabolic syndrome undergoing 18 months of diet and exercise counseling, 19 participants (38.0%) achieved remission [54]. Of 812 detected serum features, 61 metabolites were identified, and 17 changed significantly at both 12 and 18 months. A model combining prostaglandin E2, neuroprotectin D1 and taxiphyllin showed an AUC of 0.911 for distinguishing improvement in metabolic syndrome within the same small sample, without external validation. The high AUC suggests potential for identifying metabolic profiles associated with a favorable lifestyle response, which could help refine follow-up assessment. Comparison with routine clinical measures in independent cohorts would clarify whether this information improves individual risk estimates and management decisions.

### 3.4. Relating Metabolic Biomarker Patterns to Organ-Specific Immune Injury

The interpretation of metabolic biomarkers should be organized around organs in which metabolic stress becomes clinically consequential. In the liver, BCAAs, urate, ceramide species, oxidized lipids and adiponectin-related EV cargo may converge on mitochondrial dysfunction, ER stress, Kupffer-cell activation and stellate-cell fibrogenic signaling. In the kidney, hyperuricemia and glycation stress interact with reduced filtration and tubular oxidative injury, producing a feedback loop in which impaired clearance further elevates inflammatory metabolites. In the vascular system, OxLDL, Lp-PLA2, ceramides and BCAA-associated dyslipidemia influence endothelial nitric-oxide availability, leukocyte adhesion and foam-cell formation. Thus, the same metabolite concentration can have different prognostic meaning according to eGFR, liver fat, vascular imaging or myocardial injury markers [16,48,50]. This organ-aware approach reduces a common interpretive error: treating all abnormal metabolites as interchangeable measures of “metabolic inflammation.” GlycA is an integrated acute-phase glycosylation signal, urate is a purine/redox signal conditioned by renal excretion and inflammation, and an EV ceramide signature is a possible mediator of inter-organ communication. Metabolic biomarker panels should also account for treatment effects. Statins, PCSK9-directed therapy and dietary changes alter lipoprotein concentrations but may affect inflammatory metabolites differently [33,55]. Sodium-glucose cotransporter-2 inhibitors, GLP-1 receptor agonists and weight-loss interventions can alter urate handling, substrate oxidation, hepatic fat and systemic inflammation [56,57]. A fall in triglycerides may therefore not track a parallel reduction in GlycA, BCAAs or EV lipid cargo. The appropriate outcome for a novel marker is not simply correlation with an existing treatment response; it is the added ability to identify residual immune injury in patients whose conventional laboratory targets appear controlled [58]. Human tissue associations among PCSK9, adipose LDL receptor/CD36 and NLRP3 further suggest that the metabolic and inflammatory effects of lipid-lowering targets may differ [59]. Analytically, targeted mass spectrometry and NMR offer complementary paths to implementation. NMR-derived GlycA and lipoprotein insulin-resistance scores are scalable, whereas targeted LC–MS can quantify BCAAs, TMAO precursors, ceramides, oxidized lipids and selected eicosanoids with molecular specificity. Untargeted metabolomics is valuable for discovery but requires external validation, batch correction and transparent feature identification before clinical translation.

## 4. Microbiome, Barrier and Immunometabolite Alterations

**Figure 1:**
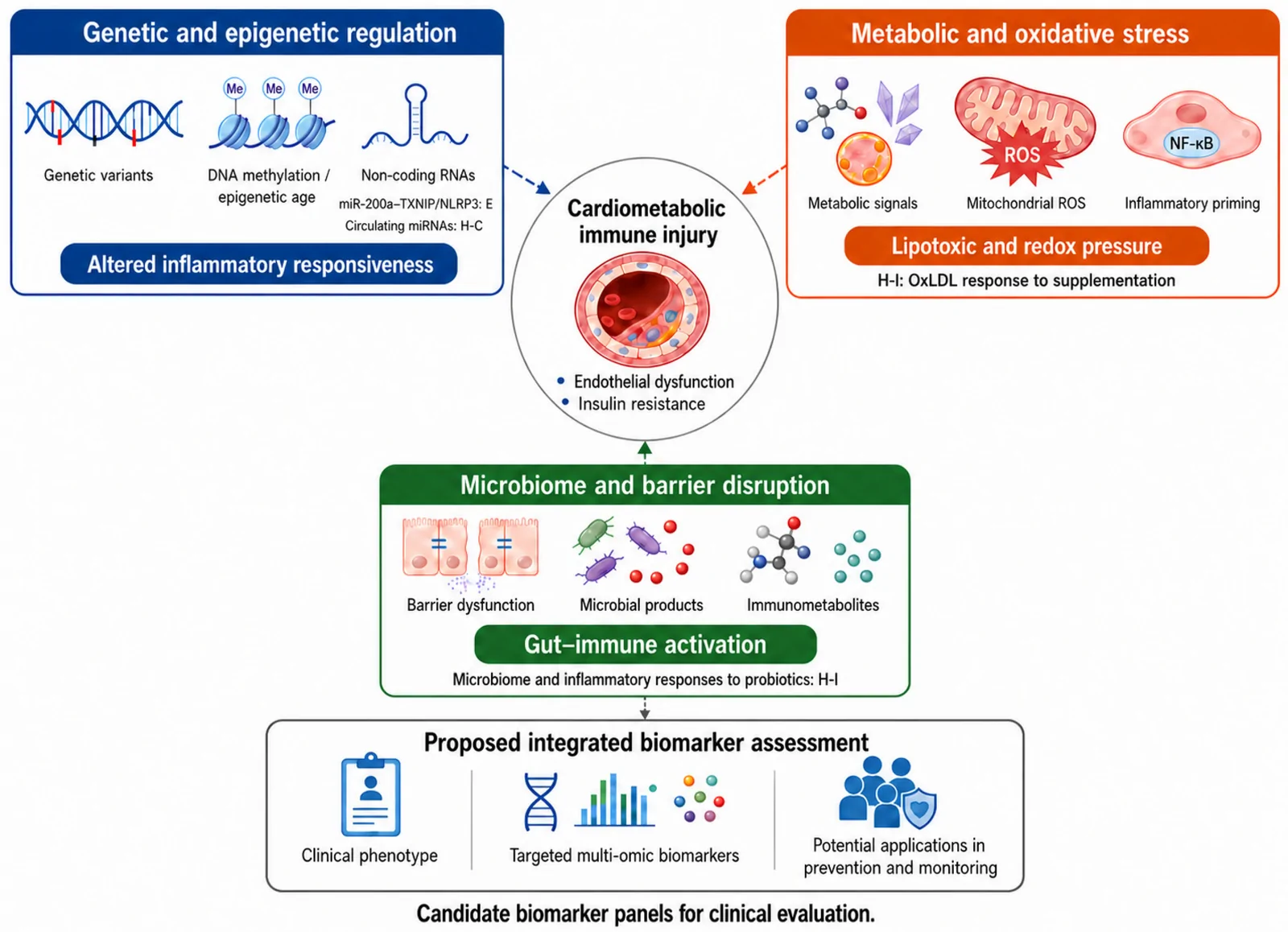
**Genetic, metabolic and microbial influences on cardiometabolic immune injury.** The schematic relates genetic and epigenetic regulation, metabolic and oxidative stress, and microbiome and barrier disruption to cardiometabolic immune injury. Dashed arrows indicate proposed links within this integrative framework. Study labels identify evidence for the adjacent examples, rather than for entire pathways or the proposed biomarker panel. The lower panel outlines candidate integration of clinical phenotype and targeted multi-omic measurements for evaluation; biomarker responses to intervention do not establish clinical benefit or the utility of biomarker-guided care. Study labels: E, experimental studies, including cellular, animal and human-derived functional experiments; H-C, human cross-sectional studies; H-I, human intervention studies. These labels describe study design and are not a formal evidence-quality grading system. Abbreviations: DNA, deoxyribonucleic acid; RNA, ribonucleic acid; Me, methyl group; miR/miRNA, microRNA; TXNIP, thioredoxin-interacting protein; NLRP3, NLR family pyrin domain containing 3; ROS, reactive oxygen species; NF-κB, nuclear factor κB; OxLDL, oxidized low-density lipoprotein.

Microbiome and intestinal-barrier changes represent the host–microbial component of this framework. Dysbiosis, altered substrate fermentation, impaired mucosal tight junctions and changed bile-acid or choline metabolism are features of altered host–microbial interactions in cardiometabolic disease. They expose lamina-propria macrophages, intestinal epithelial cells, liver Kupffer cells and circulating monocytes to LPS, peptidoglycan fragments and microbial metabolites. Barrier-associated assays and microbial metabolites provide complementary measurements of host–microbial interactions. Their interpretation must be tissue-aware: a raised LBP may reflect barrier-associated innate exposure, while TMAO also depends on hepatic FMO3 activity and kidney clearance [16,22,60]. A study of the dietary microbiota index and cardiometabolic and inflammatory markers in adults with obesity provides cross-sectional evidence. The role of the gut microbiota in cardiovascular disease has also been reviewed, but the causal chain still requires mechanistic and interventional validation [61,62]. Genetic, metabolic and microbial influences on cardiometabolic immune injury are summarized in Figure 1.

### 4.1. Barrier Dysfunction and Microbial Translocation

The gut microbiome contributes to cardiometabolic immune injury by regulating nutrient metabolism, intestinal permeability and exposure to microbe-associated molecular patterns. Dysbiosis, reduced fiber fermentation, altered bile-acid transformation and barrier dysfunction may permit lipopolysaccharide (LPS), peptidoglycan fragments and microbial metabolites to access portal and systemic circulation. These signals activate monocytes, macrophages, endothelial cells and hepatocytes, promoting Toll-like receptor signaling, NF-κB activation, cytokine production, insulin resistance and vascular dysfunction. Consequently, LPS, lipopolysaccharide-binding protein (LBP), soluble CD14 (sCD14), zonulin and markers of microbial translocation provide clinically relevant evidence that the host–microbial interface has become a source of immune injury [22,60,63]. In older adults with overweight or obesity, cardiometabolic disease and mobility limitation, Kavanagh et al. measured microbial-translocation biomarkers including LBP and sCD14 and related them to inflammation and physical function [60]. The relationship between leaky-gut markers and reduced function extends their relevance beyond biochemical profiling. In 238 young adults stratified by cardiometabolic-risk-factor burden, zonulin, LPS and CRP were jointly evaluated with diet; the findings suggested that intestinal-permeability dysfunction and low-grade inflammation coexist with adverse cardiometabolic profiles at an early life stage [63]. Commercial zonulin ELISAs have insufficient target specificity, so this signal alone cannot establish increased intestinal permeability. Thus, a person with central adiposity, dyslipidemia and elevated LPS/LBP/sCD14 or this immunoassay signal may represent a biologically distinct phenotype in which host–microbe-interface injury contributes to risk [22]. Postprandial assessment may be informative because dietary fat can transiently increase endotoxin exposure. In metabolic syndrome, a meal challenge increased postprandial endotoxin, triglycerides and incretin responses, but a two-week milk-fat-globule-membrane-enriched beverage intervention did not improve endotoxin or metabolic responses to the standardized meal challenge [64]. Such negative intervention results are still valuable: they indicate measurable endotoxin-associated metabolic stress but also show that short nutritional manipulations may not correct the underlying barrier phenotype. Accordingly, barrier markers should be interpreted with fasting/postprandial status, renal function, medications and conventional inflammatory signals.

### 4.2. Microbial Metabolites and Named Microbial Taxa Associated with Metabolic Biomarkers

Microbial metabolites provide a functional link between community composition and host cardiometabolic biology. Trimethylamine N-oxide (TMAO), generated after microbial conversion of dietary choline and carnitine followed by hepatic oxidation, is associated with insulin resistance, endothelial inflammation and cardiometabolic risk. In the Multiethnic Cohort Adiposity Phenotype Study of 1653 adults aged 60 to 77 years, median TMAO was 3.05 μmol/L [16]. Higher TMAO and carnitine and lower betaine were associated with greater insulin resistance, while choline was associated with higher systolic blood pressure, triglycerides and LBP and lower HDL cholesterol. TMAO was associated with 13 bacterial genera, including *Prevotella*, *Mitsuokella*, *Fusobacterium*, *Desulfovibrio* and genera within the *Ruminococcaceae* and *Lachnospiraceae* families. An association was also reported with the methanogen *Methanobrevibacter smithii*. A case–control study examined DASH diet adherence in adolescents with obesity and normal-weight controls in relation to metabolic syndrome and inflammatory measures, but its design cannot demonstrate benefits from dietary change [65]. These named taxa illustrate why metabolic and microbiome biomarkers should be interpreted together. For example, a profile of elevated TMAO, carnitine, LBP and insulin resistance may reflect differences in diet, microbial metabolism and host clearance but does not distinguish increased production from reduced clearance or establish a specific barrier defect. Desulfovibrio and Fusobacterium have been implicated in inflammatory ecological states in multiple settings, whereas Ruminococcaceae and Lachnospiraceae contain metabolically diverse taxa that can participate in carbohydrate fermentation and short-chain-fatty-acid production; these taxa should therefore not be classified as uniformly harmful or protective. Rather, their meaning depends on strain function, substrate availability, host diet and associated metabolites [16].

Short-chain fatty acids (SCFAs)—acetate, propionate and butyrate—provide a contrasting class of microbial metabolic signals. They can support epithelial energy metabolism, influence enteroendocrine hormone release and regulate immune-cell differentiation. In individuals at increased cardiometabolic risk, an oat-rich low-gluten diet increased fasting plasma SCFAs without significant changes in fecal microbiome composition or inflammatory markers [66]. This shows that functional metabolite readouts may respond to substrate availability even when stool taxonomic abundance appears stable. In female adolescents, central-adiposity measures and dietary intake correlated with SCFA concentrations. A study in aging men with benign prostatic hyperplasia examined prostate IL-6/IL-18 expression in relation to MetS, lipid parameters and microbiota-derived SCFAs; these tissue findings should not be generalized to circulating biomarker performance [67,68]. A single SCFA value is therefore unlikely to function as a universal prognostic marker; metabolite patterns should be assessed with diet, adiposity, barrier biomarkers and cytokine activity. A randomized study of barley products and probiotics in healthy participants also examined changes in gut hormones, metabolic measures and inflammation [69].

### 4.3. Microbiome-Targeted Interventions and Functional Interpretation

Probiotic and synbiotic trials show that microbiome-related markers can serve as intermediate response measures. In the PROSIR randomized crossover trial, *Lactobacillus reuteri* V3401 modified the gastrointestinal microbiome and improved selected inflammatory parameters in adults with metabolic syndrome [70]. The protocol for the same trial described prespecified assessments of intestinal microbiota, insulin resistance, cardiovascular risk and liver steatosis [71]. A separate synbiotic randomized trial also reported increased peptide YY in metabolic syndrome [72]. These interventions support target engagement but do not establish that microbiome change itself reduces future cardiovascular events. A randomized almond-snacking trial reported improvements in intestinal inflammation and lipid measures in adults with metabolic syndrome; a human goldenberry study suggested that oxidative-stress and permeability measures can change without parallel reductions in systemic inflammation [73,74]. These findings favor joint assessment of microbial function, host metabolism and barrier-related measures when evaluating an intervention.

The association between metabolic biomarkers and microbiome change should be conceptualized as bidirectional. Insulin resistance and obesity alter bile-acid pools, intestinal motility, gut oxygenation and dietary preference, which can reshape microbial communities. In turn, microbial metabolites influence GLP-1/PYY secretion, epithelial integrity, macrophage signaling and hepatic lipid metabolism. A decline in plasma SCFAs after low fiber exposure may signal reduced fermentation, but it can also reflect altered absorption or hepatic utilization. Similarly, elevated LBP with choline and triglycerides may indicate a combined barrier and dietary-lipid phenotype, not simply bacterial overgrowth. This is why stool taxonomic data should be integrated with plasma metabolites, dietary records, medication history and host inflammatory markers [16,66]. In human microvascular endothelial cells, TMAO and TNF-α induced partly overlapping response pathways, providing cellular mechanistic evidence [75].

## 5. Innate Training, Inflammasome and Complement Activation

This section considers innate immune sensing and effector responses in relation to the molecular and microbial processes discussed above. Repeated exposure of circulating monocytes, adipose-tissue macrophages, Kupffer cells, renal macrophages and vascular myeloid cells to glucose, oxidized lipids, urate, LPS or altered microbial metabolites induces durable changes in glycolysis, mitochondrial function and chromatin accessibility. This state of innate training lowers the threshold for NLRP3 assembly, IL-1β/IL-18 maturation and complement-associated vascular injury. Accordingly, NLRP3, ASC/caspase-1-related extracellular-vesicle cargo, IL-1β, IL-18, PTX3 and C3/C4 are candidate indicators of innate immune activity; their circulating levels alone do not establish trained immunity or identify the affected tissue [10,76]. Reviews of trained immunity in atherosclerosis and of NLRP3 in cardiometabolic disease provide context for this mechanistic framework, while the clinical validity of each biomarker still requires assessment [77,78].

### 5.1. Complement, Pentraxin-3 and Innate Effector Readiness

Complement C3 and C4 represent a distinct branch of innate immune regulation. C3 is positioned at the convergence of classical, lectin and alternative pathways, and complement cleavage products can amplify leukocyte recruitment and endothelial inflammation. In 6312 adults in Tianjin, C3 had an optimal metabolic-syndrome cutoff of 101.0 mg/dL and an AUC of 0.68, exceeding leukocyte, neutrophil and lymphocyte counts [25]. In 529 adolescents, C3, C4, CRP, fibrinogen and leptin were associated with high cardiometabolic risk, while low muscular fitness plus an adverse inflammatory profile identified the poorest metabolic risk score [79]. These data support complement as a component of multivariable phenotyping, not as an autonomous diagnostic test. In a 10-year study of 2662 adults without diabetes, adding C3 to the study model modestly improved discrimination of incident type 2 diabetes (AUC 0.609 to 0.628; *p* = 0.01) [80]. Long pentraxin-3 (PTX3) is produced by several tissue and immune-cell types and may provide information distinct from liver-derived acute-phase proteins. PTX3 distinguished mild from severe metabolic syndrome at 10.7 ng/dL, with AUC 0.656, sensitivity 47.1% and specificity 78.9% [26]. These findings suggest that C3 and PTX3 may complement routine assessment by providing additional information on the inflammatory features of MetS. The cross-sectional findings concern existing disease and its severity, rather than prediction of future cardiovascular events. In 240 adults across CKM stages, PTX3 was higher at CKM stage 2 than in controls and differentiated CKM stages 2 and 3; it also correlated with hsCRP, glycated hemoglobin and myocardial- and renal-injury markers [9]. PTX3 deficiency protected high-fat-fed mice from weight gain and adipose deposition, further supporting a mechanistic role in adipose inflammatory remodeling [81]. Together, these markers provide complementary information about innate immune and inflammatory processes, although circulating measurements do not by themselves localize tissue activity.

### 5.2. Integrated Innate-Sensing Models and Therapeutic-Response Biomarkers

Innate biomarkers may provide information about immune activity alongside measures of metabolic burden. A single elevated complement concentration is not sufficient evidence of complement-driven disease because C3 is influenced by hepatic acute-phase synthesis and adiposity [10,26]. The measured biomarker should therefore be selected for the clinical question and interpreted according to the assay and sampled compartment. Evidence of inflammasome activation requires more than component abundance, and circulating C3/C4 or PTX3 concentrations do not establish the location or cause of inflammation [10,82].

Therapeutic perturbation supports this distinction. In healthy volunteers, a three-day isocaloric ketogenic diet increased β-hydroxybutyrate and FGF21, improved insulin sensitivity and reduced ATP- or palmitate-stimulated macrophage IL-1β and TNF-α secretion; β-hydroxybutyrate and FGF21 together reduced IL-1β more than either alone in ex vivo experiments [83]. In experimental diet-induced obesity, pharmacologic NLRP3 inhibition reduced inflammatory mediators, insulin resistance, hyperglycemia and hepatic steatosis [76]. Another NLRP3 inhibitor reduced LPS/ATP- and LPS/MSU-triggered pyroptosis by approximately 66% and 62%, respectively, and improved cardiac functional injury biomarkers in high-fat-fed rats [84]. These studies do not provide clinical risk thresholds, but they show that serial innate biomarker assessment can serve as a target-engagement measure in trials. Experiments with solanesol in animal models of obesity also linked improvements in adipose macrophage inflammation to NLRP3 inhibition [85].

### 5.3. NLRP3 Inflammasome Activation as a Context-Dependent Convergence Node

The NLR family pyrin domain containing 3 (NLRP3) inflammasome is a recurrent, context-dependent convergence node through which saturated fatty acids, cholesterol crystals, extracellular ATP, mitochondrial oxidant stress, lysosomal damage and hyperglycemia can be converted into inflammatory signaling. Following priming of inflammasome components, an activating stimulus can induce NLRP3 assembly with ASC and pro-caspase-1. This promotes caspase-1 activation, maturation of IL-1β and IL-18 and, in specific contexts, gasdermin-D-mediated pyroptosis. NLRP3, caspase-1, ASC-containing extracellular vesicles, IL-1β and IL-18 constitute a mechanistically linked biomarker axis across adipose tissue, liver, kidney and vascular wall. However, NLRP3 should not be characterized as the universal common final effector molecule of cardiometabolic disorders because complement activation, nuclear factor-κB signaling, endothelial dysfunction, interferon pathways, adaptive immunity, fibrosis and thrombosis can contribute independently or in parallel [10,76]. Notably, a human liver-tissue study found no significant association of hepatic NLRP3 abundance or circulating IL-1β with insulin resistance or liver histology, suggesting that mechanisms identified in animal models do not apply uniformly to patients [86]. In a 12-week study, EPA and DHA supplementation at 2.7 g/day abolished baseline differences in adipose-tissue IL-1β secretion between the high-apoB and low-apoB groups, improved β-cell function and postprandial fat metabolism, and prevented LDL after supplementation from triggering adipose IL-1β secretion [58]. This identifies the NLRP3/IL-1β axis as a responsive signal of atherogenic-lipoprotein-driven metabolic inflammation. Moreover, large EVs carrying NLRP3 from metabolic-syndrome patients correlated with obesity- and insulin-resistance-related risk factors and promoted endothelial permeability, monocyte transmigration, smooth-muscle-cell migration and macrophage inflammatory secretion [10]. In advanced plaques, NLRP3-carrying EV accumulation and reversal of these vascular effects by NLRP3 inhibition support their potential as markers of active vascular immune injury. Renal tissue studies provide evidence of local inflammasome involvement in diabetic kidney disease. In renal-biopsy transcriptomic data from patients with diabetic kidney disease, higher connexin-43 and NLRP3 expression correlated with lower GFR and greater proteinuria; in cell experiments, connexin-43 hemichannel-mediated ATP release promoted NLRP3 activation and epithelial-to-macrophage crosstalk [82]. Connexin-43 hemichannel blockade also attenuated NLRP3-associated inflammation and tubular injury in mice with unilateral ureteral obstruction [87]. In diabetic mice, IL-22 ameliorated renal injury and fibrosis by suppressing the NLRP3/caspase-1/IL-1β pathway [88]. These findings support evaluating NLRP3-associated markers alongside albuminuria and eGFR trajectories but do not establish that circulating levels reflect renal inflammasome activity. A study in 44 hemodialysis patients associated serum NLRP3 levels with atherogenic lipid measures, providing another renal-disease context; uremia and dialysis-related confounding limit extrapolation to early diabetic kidney disease [89].

## 6. Trained Myeloid Cells and Adaptive Immune Regulation

Persistent innate training changes antigen presentation and the cytokine milieu and thereby remodels adaptive immunity. Innate and adaptive immunity interact through reciprocal feedback, with effects that vary across tissues [19,90]. Their biomarker relevance lies in measuring the cellular memory and effector readiness that connect chronic metabolic stress to continuing organ damage.

### 6.1. Metabolic Training of Innate Cells and the Bridge to Adaptive Immune Regulation

Metabolic overload can create lasting changes in immune-cell responsiveness, often described as trained immunity. In monocytes and macrophages, hyperglycemia, oxidized lipoproteins, LPS exposure, cholesterol crystals and adipose-derived cytokines can induce persistent glycolytic, mitochondrial and epigenetic reprogramming. These changes enhance secondary cytokine release and antigen-presenting capacity even after the initiating stimulus has resolved [35]. Candidate measures related to myeloid activation and training include monocyte phenotype, sCD14, sCD163, soluble TREM-related signals, EV cargo, chromatin marks, IL-1β responsiveness and monocyte-to-HDL ratio. Persistent changes in responses to restimulation, cellular metabolism or chromatin provide more direct evidence of training than soluble markers or blood-cell ratios alone. Yet the clinically important downstream consequence is frequently adaptive: antigen-presenting cells condition T-cell polarization, B-cell activation and tissue-specific immune memory [10,91]. A study of efferocytosis in central obesity involving phosphatidylserine, ADAM17, TNF-α and soluble MER provides a candidate mechanism of impaired inflammatory resolution distinct from inflammasome activation [92]. The transition from innate training to adaptive activation is relevant in adipose tissue, liver, vascular wall and kidney. Activated macrophages and dendritic cells produce IL-1β, IL-6, IL-12, IL-18 and TNF-α while presenting modified lipids, oxidized neoepitopes or damage-associated molecules to T cells. This environment favors Th1 and Th17 polarization, suppresses regulatory T-cell (Treg) function and supports B-cell antibody production [90,93]. Consequently, emerging adaptive immune biomarkers include the relative abundance and activation status of CD4+ Th1, Th17 and Treg cells; cytotoxic CD8+ T-cell activation; circulating memory B-cell or plasmablast phenotypes; autoantibodies to oxidized lipoproteins; T-cell receptor repertoire features; immune-cell transcriptomic modules; and cytokines such as IL-17, IL-21, IL-10 and transforming growth factor-β. Although many are not yet standardized clinical tests, they may help characterize immune phenotypes not captured by routine metabolic measures.

S100A8/A9 (calprotectin) is an alarmin released by activated neutrophils and monocytes [94]. Experimental studies show that it can impair endothelial barrier function through TLR4 and RAGE signaling [95]. In prospective cohorts of patients with acute myocardial infarction, higher circulating S100A8/A9 was associated with subsequent heart failure, with consistent findings in discovery and validation cohorts [96]. These experimental and observational findings support its potential for cardiovascular risk stratification, but clinical benefit from S100A8/A9-guided management has not been established.

### 6.2. T-Cell Polarization and Adaptive Immune Injury Phenotypes

A Th1-skewed immune state, characterized by interferon-γ and TNF-α signaling, can intensify macrophage activation and insulin resistance. Th17 cells produce IL-17A/F and promote endothelial activation, chemokine expression and neutrophil recruitment, whereas Tregs limit inflammatory activation through IL-10, TGF-β and contact-dependent suppression [19,90,97]. The balance of Th17 to Treg cells is therefore a plausible biomarker of unresolved cardiometabolic inflammation. In obesity, IL-17E and IL-17F correlated positively with BMI, and IL-17E rose with subcutaneous fat; higher IL-17E was observed among participants reporting atherosclerosis and/or beta-blocker use [98]. These serum associations do not establish a Th17 cell phenotype; IL-17E also participates in type 2 immune responses. Experimental and human work also identifies IL-17 as a cytokine linking psoriasis and hyperglycemia, supporting the idea that adaptive immune dysregulation can contribute to metabolic disease across tissue contexts. Research on AKR1B10, retinol metabolism and IL-17 signaling in psoriasis provides a cross-disease immunometabolic clue [99]. Adaptive immune regulation should be assessed as a network rather than a static cell count. A reduced Treg compartment or elevated Th17/Treg ratio may indicate loss of resolution capacity, whereas elevated activated CD8+ T cells may signal cytotoxic stress in adipose, liver or vascular tissue [20,100].

### 6.3. B-Cell, Antibody and Immune-Glycomic Biomarkers

B cells can contribute to cardiometabolic inflammation through antigen presentation, cytokine production and antibody-mediated activation of Fc receptors and complement [93,101]. IgG glycosylation is particularly informative because it provides a functional output of humoral immune regulation [102]. The modest but significant discrimination of metabolic syndrome and abdominal obesity by IgG N-glycan traits demonstrates that antibody effector phenotype can track cardiometabolic status [24]. Reduced anti-inflammatory glycan features may enhance Fcγ-receptor engagement and complement activation, thereby linking adaptive immunity to endothelial injury and tissue inflammation. Autoantibodies against oxidized lipoproteins, phospholipids or other damage-associated antigens have been proposed as related indicators, but their directionality can vary because some natural antibodies facilitate clearance whereas others intensify inflammation [101]. Therefore, the clinical priority is not simply to measure antibody titre but to interpret antibody specificity, subclass, glycosylation and complement-fixing capacity. Integration of B-cell phenotyping, IgG glycomics, complement activation and oxidized-lipid biomarkers could reveal an adaptive-humoral endotype of cardiometabolic risk. This approach remains investigational but is conceptually aligned with precision medicine: immune-cell programming is likely heterogeneous even among patients with similar HbA1c, blood pressure and lipid values.

### 6.4. From Cell Phenotypes to Clinically Feasible Adaptive Immune Biomarker Panels

The main obstacle to adaptive immune biomarker translation is that cell states are more informative than total lymphocyte number, yet detailed phenotyping is technically demanding. Conventional flow cytometry can quantify activated CD4+ and CD8+ T cells, Tregs, Th17-like populations, memory B cells and expression of markers such as HLA-DR, CD38, PD-1, CCR6 or CXCR3 [23]. Functional assays can measure cytokine production after stimulation, whereas single-cell RNA sequencing can map exhaustion, cytotoxicity, interferon response, antigen presentation and metabolic programs [100]. The appropriate platform depends on the purpose: broad clinical screening requires low-cost reproducible assays, while discovery studies may use deep phenotyping to derive a smaller clinically deployable signature. Adaptive markers must also be interpreted in tissue context. Peripheral blood may underrepresent pathogenic immune cells retained in visceral adipose tissue, plaque, kidney or liver [100]. Nevertheless, blood-based signatures can be valuable if they correlate with a relevant tissue phenotype or longitudinal outcome.

For cellular assays, chronic viral infection, recent vaccination and immunomodulatory treatment warrant particular attention because they can alter the measured immune subsets. Inflammatory mediation analyses of sleep and cardiovascular–kidney–metabolic stages, cytokine studies in obstructive sleep apnea, longitudinal Whitehall II analyses of work stress and analyses of alcohol-consumption trajectories suggest that non-metabolic exposures can alter inflammatory measures; statistical mediation alone does not prove causal mediation [103,104,105,106]. Research on sleep-disordered breathing in children with overweight or obesity also linked CRP to its severity, with cardiorespiratory fitness potentially modifying this relationship [107].

## 7. Cytokine Release, Hematologic Indices and Complement Effectors

**Figure 2:**
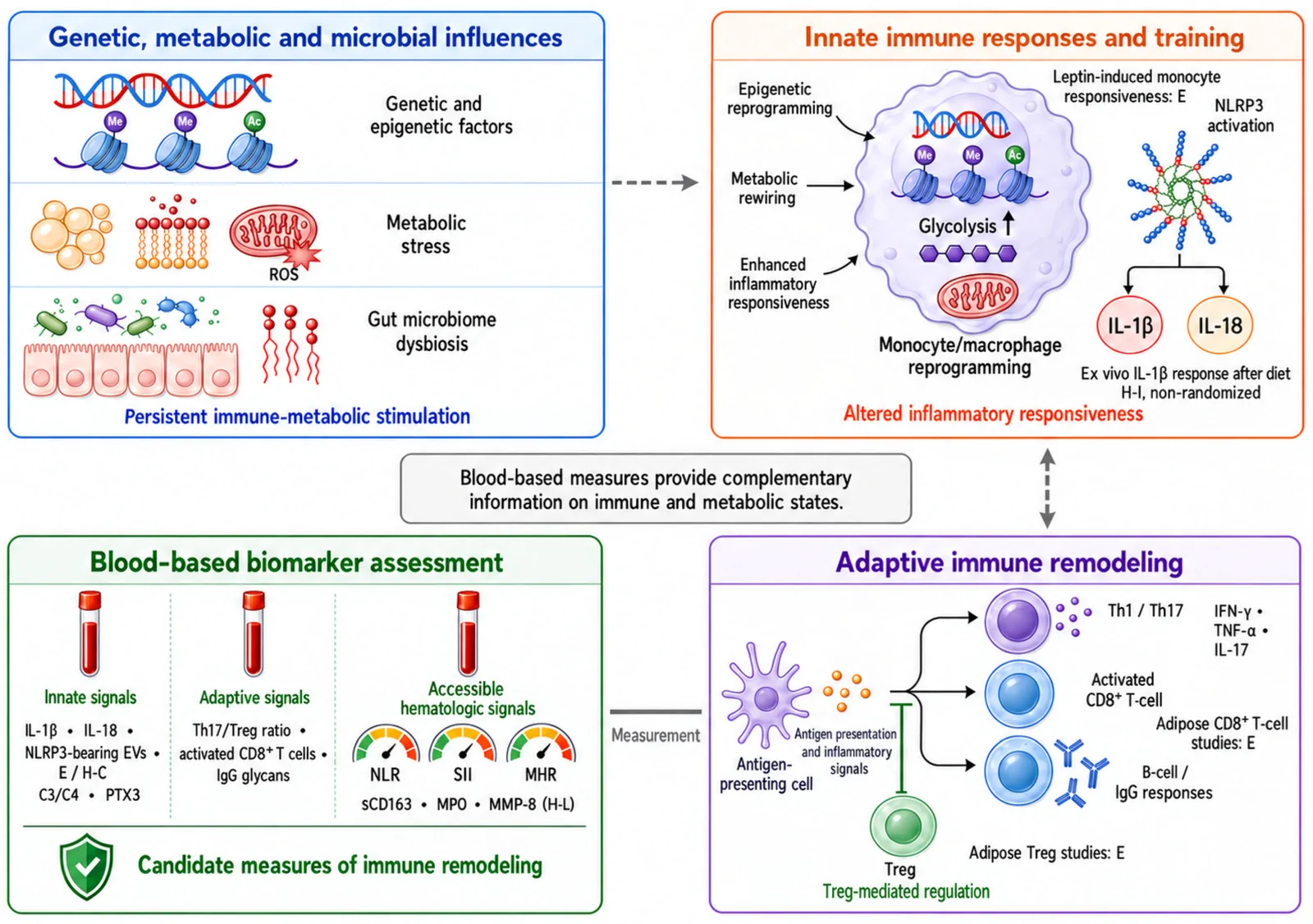
**Immune responses and blood-based biomarker assessment in cardiometabolic disorders.** The schematic connects genetic, metabolic and microbial influences with innate and adaptive immune responses and candidate blood-based measures. Internal arrows depict selected cellular mechanisms, dashed arrows indicate proposed interactions, the gray line denotes measurement, and the green inhibitory symbol represents Treg-mediated regulation. Circulating markers provide complementary information but do not alone establish trained immunity or tissue origin. Study labels describe the adjacent examples: E, experimental; H-C, human cross-sectional; H-L, human longitudinal observational; H-I, human intervention. They indicate study design rather than evidence quality or clinical utility. Abbreviations: Me, methyl group; Ac, acetyl group; ROS, reactive oxygen species; NLRP3, NLR family pyrin domain containing 3; IL, interleukin; Th1/Th17, type 1/type 17 T helper cells; Treg, regulatory T cell; CD8, cluster of differentiation 8; IgG, immunoglobulin G; IFN-γ, interferon-γ; TNF-α, tumor necrosis factor-α; EVs, extracellular vesicles; C3/C4, complement components 3 and 4; PTX3, pentraxin 3; NLR, neutrophil-to-lymphocyte ratio; SII, systemic immune-inflammation index; MHR, monocyte-to-high-density lipoprotein cholesterol ratio; sCD163, soluble cluster of differentiation 163; MPO, myeloperoxidase; MMP-8, matrix metalloproteinase-8.

Circulating cytokines, blood-cell indices and complement provide accessible measures of systemic immune activity and are considered here in relation to the molecular and cellular processes discussed above. Immune remodeling and blood-based readouts are shown in Figure 2 [10,12,14]. In prospective cohorts, inflammatory markers and cardiovascular–kidney–metabolic stages were jointly associated with mortality. Research integrating CRP, TyG and visceral adiposity indices provides risk-marker associations; incremental value requires testing in independent samples [108,109].

### 7.1. Cytokine Networks as Mechanistic and Prognostic Readouts

Cytokine biomarkers capture the inflammatory communication through which metabolic stress becomes tissue injury. IL-1β and IL-18 are inflammasome-linked mediators; TNF-α and IL-6 connect adipose, oxidative and ER stress to insulin resistance; MCP-1 recruits monocytes; IL-17 and IL-32 can report lymphocyte-driven or amplification phenotypes; and IL-10 and IL-22 represent regulatory or tissue-protective capacity. Their interpretation depends on the cytokine, assay and clinical context [14,98,110]. Studies of less-established cytokines illustrate the gap between biological plausibility and clinical usefulness [111,112]. Tissue-expression studies provide additional biological context but require separate evaluation as circulating tests [113]. Associations among lipids, CRP and cytokines in healthy adults suggest that information about subclinical inflammation also depends on baseline health [114]. IL-1β drives vascular inflammatory programming under hyperglycemic conditions. In human aortic smooth-muscle cells, IL-1β activated NLRP3, shifted metabolism toward glycolysis and pentose-phosphate-pathway activity, and promoted release of small EVs enriched in pro-caspase-1; high glucose amplified these responses, while anakinra prevented inflammasome activation, metabolic reprogramming and EV release [115]. These experiments support a role for IL-1β signaling in the response of vascular smooth-muscle cells to high glucose. TNF-α is an integrator of metabolic and ER stress. LPS, palmitate and oleate acted with ER stress to increase TNF-α, ROS, CHOP, ATF6 and NF-κB signaling in monocytes, while antioxidants and pathway inhibitors attenuated TNF-α production [110]. Participants with obesity had higher adipose and plasma TNF-α together with CRP, malondialdehyde and oxidized LDL [110]. IL-6 provides complementary information about visceral adiposity and residual inflammation. In a young-adult birth cohort, the number of chronic diseases was directly associated with IL-6 and CRP in men at 22 years, whereas multimorbidity was inversely associated with adiponectin in women [116]. These results support sex-specific models and demonstrate why cytokines should be interpreted with body composition, adipokines, renal function and metabolic measures. Serum IL-6 was associated with visceral fat mass in older adults, while experiments in aged mice further examined its role in lipolysis [117]. Among outpatients with at least one metabolic-syndrome component, CRP was associated with epicardial and visceral fat depots in women [118]. Cross-sectional associations among dietary fat, leptin and cytokines provide nutritional context [119]. Experiments with postprandial triglyceride-rich lipoproteins in THP-1 macrophages suggested that olive-oil-derived oleanolic acid can influence IL-6 responses [120]. MCP-1/CCL2 marks monocyte recruitment. In type 2 diabetes, omega-3 PUFA supplementation improved MCP-1 and lipid profile without changing resistin, showing that chemokine regulation can be partly independent of other adipose immune mediators [121]. Other cytokines have shown case–control differences and correlations with metabolic traits [122]. Regulatory cytokines add context: IL-10 may indicate compensatory suppression but can also reflect greater upstream inflammatory pressure. Cross-sectional data in Indonesian adults associated central obesity with hsCRP but found no significant hsCRP difference between omega-3 intake groups [123].

### 7.2. Hematologic Indices and Monocyte/Macrophage Activation

Hematologic indices use routine blood counts to capture systemic myeloid, lymphoid and platelet imbalance. The neutrophil-to-lymphocyte ratio (NLR), systemic immune-inflammation index (SII) and monocyte-to-HDL ratio (MHR) are representative, accessible measures, but they are not disease-specific tests. Their clinical value lies in identifying a cellular inflammatory phenotype that may accompany insulin resistance, adipose hypoxia, steatosis, oxidative stress and endothelial activation [13,124,125]. Other composite ratios have also been examined, but their added value over the component measurements remains to be established [126,127]. A study of leukocyte counts and incident metabolic syndrome during antipsychotic treatment indicates that medication exposure and underlying disease must be included in the intended-use population [128]. SII, calculated as platelets × neutrophils/lymphocytes, has consistent population-level associations. In NHANES 2011–2016, among 12,402 adults, the highest versus lowest SII quartile was associated with metabolic syndrome (OR 1.33, 95% CI 1.14–1.55), whereas continuous log-transformed SII (Lg-SII) was associated with the elevated waist circumference component (OR 2.17, 95% CI 1.65–2.87) and the elevated blood-pressure component (OR 1.65, 95% CI 1.20–2.27) [129]. In MESA, continuous ln(SII) was associated with metabolic syndrome, hyperglycemia and elevated blood pressure, and the highest versus lowest quartile had adjusted odds ratios of 1.32 (95% CI 1.03–1.69) for hyperglycemia and 1.63 (95% CI 1.25–2.14) for elevated blood pressure [124]. In another NHANES analysis, CKM was operationally defined as cardiometabolic syndrome accompanied by chronic kidney disease; each 1000-unit increase in SII was associated with an OR of 1.48 (95% CI 1.20–1.81) for this outcome [130]. These observations support SII as a practical enrichment marker, while its ability to predict incident hard outcomes beyond clinical risk models still requires prospective evidence. MHR combines monocyte count with HDL cholesterol concentration but does not directly measure the balance between inflammatory and anti-inflammatory activity. In NHANES analyses of 49,929 adults, high MHR was strongly associated with diabetes (aOR 1.76) and cardiovascular disease (aOR 1.69); in 35,781 participants with mortality data, associations were highest for kidney-disease mortality (aHR 3.13) and diabetes mortality (aHR 2.26) [13]. The white-blood-cell-to-mean-platelet-volume ratio (WMR) has also been associated with prevalent metabolic syndrome [125]. Prospective UK Biobank analyses reported differences in inflammation–mortality associations by sex and glycemic status. Research on central obesity and mortality also evaluated statistical mediation by inflammatory markers, which cannot be interpreted directly as causal mediation [131,132]. Cross-sectional mediation analyses provide exploratory evidence rather than establishing causal pathways [133]. Macrophage activation markers add mechanistic specificity. In longitudinal carotid-ultrasound cohorts, sCD163 was associated with plaque formation in virally suppressed men with HIV, whereas Gal-3BP and galectin-3 did not predict plaque in that setting [134]. Gal-3BP nonetheless predicted incident diabetes, metabolic syndrome, chronic liver disease and multimorbidity in the InCHIANTI study [135]. These contrasting results show that a marker may be informative for metabolic deterioration without being a universal predictor of plaque formation.

### 7.3. Cytokine Signatures in Organ-Specific Cardiometabolic Injury

The vascular mechanisms described in Section 7.1 provide a basis for relating cytokines to vascular phenotypes, but circulating concentrations do not establish a vascular source or causal role [14,115]. The LURIC cohort compared cardiovascular risk information from hsCRP, serum amyloid A and IL-6 [136]. Human iPSC-derived endothelial models suggested sex differences in inflammatory and mitochondrial responses related to metformin and resveratrol [137]. Studies of platelet-related markers in women with obesity and simple blood-count indices in childhood obesity support pairing inflammatory measures with specific liver phenotypes. However, negative findings in type 1 diabetes with metabolic syndrome caution against assuming a uniform hepatic inflammatory signal across subgroups [138,139,140]. A retrospective analysis of ferritin, obesity and liver ultrasound changes in children with metabolic syndrome provides additional information, although ferritin is also affected by iron metabolism [141]. Paired measurements of IL-10 or IL-22 with IL-1β, TNF-α and IL-6 may help characterize an inflammatory profile but do not directly measure regulatory capacity. Likewise, relationships between cytokines and adiponectin, leptin or the adiponectin/leptin ratio may link circulating inflammation to adipose dysfunction but do not establish adipose tissue as the source. The relationship of IL-6 to diabetes and metabolic syndrome has also been examined in the ethnically diverse REGARDS cohort, indicating the importance of considering population composition alongside longitudinal outcomes [142]. In an exercise study in women with obesity, circulating IL-6 increased with exercise intensity, suggesting that exercise-associated IL-6 changes should not be equated directly with worsening chronic inflammation [143].

### 7.4. Hematologic Indices as Longitudinal Risk-Enrichment Tools

Hematologic indices are attractive because they are available in routine care, but their biological meaning derives from the components rather than the arithmetic formula. A high NLR can arise from neutrophilia, lymphopenia or both. Neutrophilia may reflect acute or chronic innate activation and degranulation, whereas lymphopenia can indicate stress-hormone effects, immunosenescence, malnutrition or altered adaptive regulation. In nascent metabolic syndrome, neutrophil count was reported to be superior to NLR as a marker of inflammation, illustrating the need to inspect component counts rather than relying solely on the ratio. Similarly, SII may be elevated through platelets, neutrophils or lymphocytes, each of which has distinct implications for thrombosis, innate activation and adaptive immune balance [124,144]. Evidence in children with obesity and metabolic syndrome links NLR and SII to macular retinal damage, demonstrating that hematologic indices can be associated with a microvascular target-organ phenotype. In men with erectile dysfunction, NLR greater than 3 was associated with severe disease, another clinical phenotype in which vascular and inflammatory mechanisms intersect [125,145,146]. Longitudinal change deserves particular attention. High-dose vitamin-D supplementation reduced hsCRP and shifted the distribution of NLR toward lower values, while standard treatment of prepubertal obesity produced favorable changes in HOMA-IR, IL-6, CRP, NLR and platelet-to-lymphocyte ratio (PLR) alongside reductions in alanine aminotransferase [147,148]. These observations do not prove that changing NLR causes clinical benefit, but they suggest that serial indices may act as accessible pharmacodynamic measures when they move coherently with metabolic and tissue-injury endpoints. Hematologic disorders, corticosteroid exposure and pregnancy can also alter component counts and require attention in the intended population. In a choline-containing egg intervention, IL-6 decreased without a parallel change in plasma cholesterol, again showing that inflammatory and conventional lipid measures need not move together [149]. Exercise training in men with metabolic syndrome also produced changes in body composition, insulin resistance and IL-8 [150].

### 7.5. Tissue Remodeling, Neutrophil Degranulation and Complement-Integrated Assessment

MMP-8, MMP-9, myeloperoxidase and resistin describe the effector phase of immune-cell activation, in which proteolysis, oxidant production and extracellular-matrix turnover translate inflammation into structural injury. In 127 metabolic-syndrome patients undergoing diet and behavioral counseling, baseline MMP-8, MMP-9 and MPO increased with the number of syndrome criteria [29]. At six months, 73 participants (57%) met fewer metabolic-syndrome criteria than at baseline, and low baseline MMP-8 independently predicted this improvement (adjusted OR for baseline MMP-8 0.970, 95% CI 0.949–0.993; *p* = 0.009); a model with MMP-8, baseline criteria and waist circumference showed apparent discrimination, and the study reported 1000 bootstrap resamples for the OR estimates [29]. The prospective association supports further evaluation of MMP-8 as an aid to identifying patients less likely to improve during lifestyle management. If confirmed in independent cohorts, this information could help prioritize closer follow-up; its added value should be assessed against the same clinical model without MMP-8. Higher baseline MMP-8 was therefore associated with a lower likelihood of metabolic improvement during follow-up. This is prognostic evidence for metabolic improvement, not evidence that MMP-8 identifies differential benefit from lifestyle intervention. A Tunisian cross-sectional study also measured matrix metalloproteinases and inflammatory mediators in metabolic syndrome [151]. Associations of mast-cell-related IgE, chymase and tryptase with metabolic syndrome in Chinese adults broaden the candidate pathways [152].

## 8. Cross-Cutting Integration Across Cardiometabolic Disorders

### 8.1. From Isolated Biomarkers to Mechanism-Defined Immune-Metabolic Endotypes

**Figure 3:**
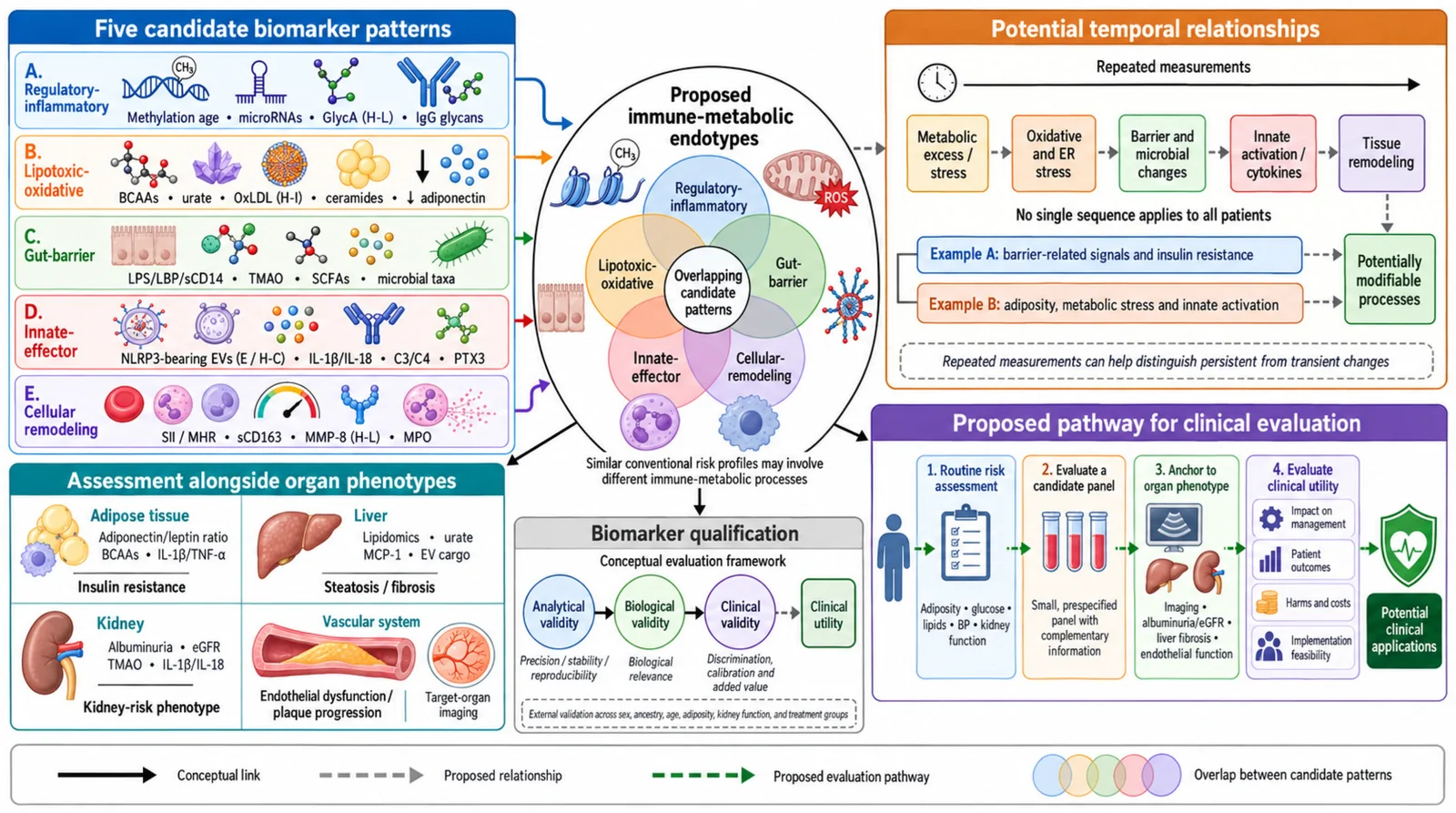
**Proposed integration of immune-metabolic biomarker patterns and clinical evaluation.** Five overlapping candidate patterns link biomarker assessment with organ phenotypes, repeated measurements and clinical evaluation. The proposed endotypes and panels require validation; the framework implies neither a uniform disease sequence nor tissue specificity of circulating signals. Biomarker qualification encompasses analytical, biological and clinical validity and clinical utility. The proposed evaluation pathway considers added predictive value, management decisions, patient outcomes and practical implementation. Study labels (E, experimental; H-C, human cross-sectional; H-L, human longitudinal observational; H-I, human intervention) apply to individual examples, not the framework as a whole. Abbreviations: CH_3_, methyl group; ROS, reactive oxygen species; GlycA, nuclear magnetic resonance signal from glycosylated acute-phase proteins; IgG, immunoglobulin G; BCAAs, branched-chain amino acids; OxLDL, oxidized low-density lipoprotein; LPS, lipopolysaccharide; LBP, lipopolysaccharide-binding protein; sCD14, soluble cluster of differentiation 14; TMAO, trimethylamine N-oxide; SCFAs, short-chain fatty acids; NLRP3, NLR family pyrin domain containing 3; EVs, extracellular vesicles; IL, interleukin; C3/C4, complement components 3 and 4; PTX3, pentraxin 3; SII, systemic immune-inflammation index; MHR, monocyte-to-high-density lipoprotein cholesterol ratio; sCD163, soluble cluster of differentiation 163; MMP-8, matrix metalloproteinase-8; MPO, myeloperoxidase; TNF-α, tumor necrosis factor-α; MCP-1, monocyte chemoattractant protein-1; eGFR, estimated glomerular filtration rate; ER, endoplasmic reticulum; BP, blood pressure.

The proposed endotype framework groups biologically related signals to help investigate differences in disease trajectories among people with similar conventional risk factors. The framework and its associated panel-based assessment pathways remain to be validated. Candidate patterns include regulatory-inflammatory signals (methylation age, microRNAs, GlycA and IgG glycans), lipotoxic-oxidative signals (BCAAs, urate, OxLDL and ceramides), gut-barrier signals (LPS/LBP/sCD14, TMAO and SCFAs), innate effectors (NLRP3-related EVs, IL-1β/IL-18, C3/C4 and PTX3), and cellular remodeling (SII/MHR, sCD163, MMP-8 and MPO). Figure 3 integrates these endotypes and temporal trajectories [16,24,27]. These endotypes overlap. Visceral adiposity can generate lipotoxic stress, prime NLRP3 and impair gut integrity; LPS and cytokine responses then interact with hepatic acute-phase glycosylation and blood-cell indices. A focused panel should begin with established clinical risk measures and add a small number of biologically relevant markers for a defined question [10,27,28].

Among the markers discussed, hsCRP, GlycA and IL-6 merit particular attention for further clinical evaluation. hsCRP offers a readily available reference measure of systemic inflammation. GlycA provides an integrated acute-phase signal with prospective associations with diabetes and cardiovascular events [27], whereas IL-6 links experimental inflammatory pathways with longitudinal human evidence [136,142]. Their practical roles differ: GlycA requires NMR-based measurement, and IL-6 testing remains less accessible than hsCRP [27,136]. These markers provide a focused starting point for evaluation, rather than an established combined panel; their added value should be assessed for a defined population and clinical outcome.

Table 2 compares selected biomarkers by biological source, clinical evidence, validation, potential use and practical limitations.

A staged approach offers a practical way to evaluate candidate panels. First-line assessment includes waist circumference, glycemia, lipids, eGFR, albuminuria, hsCRP and blood counts. An investigational second tier can target the suspected metabolic-oxidative, gut-barrier, inflammasome or cellular endotype. Epigenetic, microRNA, immune-phenotyping and metagenomic tests remain research tools until incremental value is established [10,12,16,24]. Studies of fetuin-A, YKL-40 and hsCRP, and analyses of residual inflammatory and LDL-cholesterol risk, reinforce assessment against a defined clinical use and existing models [30,154]. The AHA framework distinguishes initial proof of concept and prospective validation from incremental predictive value, changes in clinical management, improved outcomes and cost-effectiveness [34,153].

Biological complementarity should be assessed alongside correlations between candidate markers because markers assigned to different pathways may still provide overlapping information. The selection procedure should be specified before model fitting, with each addition evaluated against established clinical predictors, hsCRP and IL-6 as inflammatory comparators, and the other markers in the panel. MetS is already defined by routinely measured waist circumference, blood pressure, glucose and lipids; a biomarker that distinguishes prevalent MetS therefore does not, on that basis alone, have an additional diagnostic role. For prognostic use, prospective studies should establish whether the marker predicts incident disease or clinical events beyond these components and established risk factors, rather than merely showing an adjusted association [153]. Sample-size planning should account for the number of candidate predictor parameters, outcome frequency and anticipated model performance, rather than only the number of markers retained in the final model [155]. The resulting panel should undergo external validation for discrimination, calibration and incremental clinical value. Where clinically meaningful risk thresholds exist, reclassification should be assessed against the clinical model, with results reported separately for people with and without events. Decision-curve analysis can compare net benefit with and without the new marker across plausible decision thresholds; it does not replace evidence that test-guided care improves outcomes [34]. Standardized assays are needed for reproducible use across centers, while cost, turnaround time and availability determine whether the panel is practical. A smaller panel that retains useful complementary information may be easier to implement and more suitable for repeated assessment [34].

### 8.2. Organ-Specific Anchoring of Systemic Immune Injury Biomarkers

Cardiometabolic disorders provide a practical framework for anchoring systemic biomarkers to clinical outcomes. In adipose tissue, a low adiponectin/leptin ratio, BCAA accumulation, macrophage activation and IL-1β/TNF-α signaling can mark loss of metabolic flexibility and escalating insulin resistance. In liver disease, lipidomic signatures, urate, cytokine activation, MCP-1 recruitment and EV cargo may signal the transition from steatosis to inflammatory and fibrotic injury. In kidney disease, urate, TMAO, IL-1β/IL-18, NLRP3 activity, complement and tubular inflammatory signaling can be interpreted with albuminuria and eGFR decline. In the vascular system, OxLDL, Lp-PLA2, PTX3, complement, NLRP3-positive EVs, SII and MMP-8 can be assessed against endothelial function, plaque burden or clinical events [10,48,82]. Systemic inflammation is neither organ-specific nor uniformly harmful. High SII with albuminuria and falling eGFR suggests a different phenotype from isolated SII elevation. TMAO must be interpreted with diet and renal clearance, while IL-6 may reflect adiposity, infection, muscle signaling or local inflammation. Organ phenotypes and longitudinal trajectories sharpen their interpretation [12,14]. Clinical endophenotypes should accompany broad metabolic-syndrome definitions. Retinal associations of NLR/SII in children and lifestyle-response prediction by MMP-8 connect immune markers to organ involvement or recovery [29,145]. Pediatric NLRP3, adolescent longitudinal and childhood glycemic-variability studies highlight developmental context [156,157,158]. Soluble TNF receptors are also associated with pediatric adiposity and metabolic measures [159].

### 8.3. Methodological Considerations for Biomarker Qualification

Assay precision, stability and pre-analytical handling are especially important for cytokines, EVs, microRNAs and metabolites [10,22]. Equity and implementation matter from the outset. Ancestry, diet and access to advanced assays affect generalizability. Validation cohorts should reflect the age range, sex distribution, medication use and comorbidities of the intended population. Where sample size permits, prespecified subgroup analyses can help determine whether predictive performance is consistent across these groups [34]. Low-cost first-line indices, with molecular testing reserved for uncertain phenotypes or high residual risk, could support precision without widening disparities. Limited agreement between metabolic-syndrome classification and cardiovascular risk scores in Negrito adults reinforces target-population validation [5].

Interpretation also depends on the clinical context and sample handling. Renal function and dietary intake are particularly relevant to circulating metabolites, whereas infection, smoking, adiposity and medication use can influence inflammatory proteins and blood-cell indices. Age, sex and blood-cell composition should be considered when interpreting epigenetic and cellular signatures; diet and antibiotic exposure are relevant to microbiome-derived markers. Pre-analytical influences also differ: hemolysis can alter circulating microRNA measurements, and EV measurements depend on sample processing and isolation procedures. Sampling conditions, processing delays and storage should therefore be documented and standardized where feasible. Clinical variables should be addressed according to the intended use and study question, since some may represent confounders, mediators or consequences of disease. Accounting for these influences can improve comparability across studies and help identify biomarker signals that remain informative in routine clinical settings.

### 8.4. Dynamic Trajectories and Causal Ordering of Biomarker Change

Repeated panels can identify trajectories and potentially reversible nodes. Gut-barrier disruption and LPS exposure may lead in some patients; adipose dysfunction, BCAA accumulation and oxidative stress may lead in others. Temporal ordering need not be uniform [10,16]. Baseline hsCRP associations with hypertension remission also link inflammation to disease reversal, without establishing biomarker-guided treatment benefit [160]. Longitudinal mediation, intervention experiments and genetic instruments can clarify whether biomarkers are causes, consequences, compensatory responses or treatment-responsive correlates. Intervention studies can then test whether changes in these pathways accompany metabolic improvement, as illustrated by the diet-related functional responses discussed in Section 5.2 [83].

Human genetic evidence does not support CRP itself as a causal mediator of coronary heart disease, whereas IL6R variants support a causal role for IL-6 receptor signaling [161,162]. In CANTOS, canakinumab at 150 mg every three months reduced recurrent cardiovascular events in patients with previous myocardial infarction and elevated hsCRP, without lowering LDL cholesterol [163]. This supports IL-1β as a therapeutic target but does not directly establish the benefit of NLRP3 inhibition or biomarker-guided treatment selection. In CIRT, low-dose methotrexate did not lower IL-1β, IL-6 or CRP and did not reduce cardiovascular events [164]. In CLEAR SYNERGY, colchicine lowered CRP but did not reduce the primary cardiovascular composite outcome when initiated soon after myocardial infarction [165]. These findings show that clinical benefit depends on the targeted pathway, intervention and patient population and cannot be inferred from biomarker reduction alone.

## 9. Future Directions

Future work should test whether the proposed immune-metabolic endotypes identify differences in disease progression or treatment response. Hypothesis-driven multi-omics can connect regulatory potential, metabolic stress, host–microbial interactions and immune effectors [1,12,166]. Priorities are to identify informative signals in defined populations, establish their relationship to tissue injury and determine whether using them improves care. The focused panel-development and qualification strategies outlined in Section 8 provide a basis for this work.

## Figures and Tables

**Table 1:** **Evidence types and interpretation of selected biomarkers discussed in this** **review.**

Evidence Type	Representative Biomarkers	Evidence in Cited Studies	Interpretation and Limits	Refs.
Mechanistic	NLRP3-bearing EVs; miR-200a/TXNIP/NLRP3; adipose Treg and CD8+ T cells	Human-derived EV experiments, cell studies and animal models link these signals to inflammatory pathways or metabolic dysfunction.	Experimental perturbation supports mechanisms in the tested model, not diagnostic or prognostic performance of circulating measurements in patients.	[10,18,19,20]
Analytical	Zonulin immunoassays; blood DNA methylation; immune-cell phenotyping	Assay-specific findings question the identity of measured zonulin. Cell-mixture estimation and flow-cytometry guidance address measurement variation.	Analytical evidence concerns measurement identity and reliability. Use in a clinical association study alone does not validate an assay for its proposed clinical use.	[21,22,23]
Diagnostic	IgG N-glycans; complement C3; PTX3	Cross-sectional studies assess prevalent metabolic-syndrome discrimination or differences between severity groups, including ROC analyses.	These exploratory evaluations do not establish detection of organ-specific immune injury or prediction of future events.	[24,25,26]
Prognostic	IL-6 and hsCRP; GlycA; urate with hsCRP; SII/SIRI and MHR; MMP-8	Longitudinal studies associate baseline measurements with subsequent metabolic outcomes, cardiovascular events or mortality. MMP-8 was associated with fewer metabolic-syndrome criteria at follow-up.	Outcome association, incremental prediction and external validation are distinct. Follow-up in an intervention cohort does not establish differential treatment benefit.	[12,13,14,27,28,29,30]
Predictive of treatment benefit	MMP-8; OxLDL/Lp-PLA2; selected sphingolipids	Studies report metabolic improvement after counseling, biomarker changes during randomized supplementation, or selected sphingolipid changes before and after Ramadan fasting. Ceramides and dihydroceramides did not change significantly in the fasting study.	Treatment effects must be compared across biomarker-defined groups. The cited response observations do not establish differential benefit or support treatment selection.	[29,31,32]
Clinical utility	hsCRP in JUPITER; proposed immune-biomarker panels	JUPITER demonstrated rosuvastatin benefit in adults selected for elevated hsCRP and other eligibility criteria. Clinical implementation requirements are also discussed.	A biomarker-selected trial does not directly compare test-guided care with usual care. Proposed panels require evidence of improved decisions and patient outcomes.	[33,34]

The categories describe different evidence questions and are not interchangeable stages of validation. A biomarker may contribute to more than one category. “Prognostic” groups longitudinal outcome evidence here, including incident disease and intermediate metabolic outcomes; it does not imply a validated clinical risk model. The predictive and clinical-utility rows identify relevant examples and remaining gaps, not established uses. This table covers selected studies already cited in the review and is not an exhaustive assessment of every biomarker. Abbreviations: EV, extracellular vesicle; Treg, regulatory T cell; ROC, receiver operating characteristic; hsCRP, high-sensitivity C-reactive protein; SII, systemic immune-inflammation index; SIRI, systemic inflammation response index; MHR, monocyte-to-HDL ratio; OxLDL, oxidized LDL; Lp-PLA2, lipoprotein-associated phospholipase A2; MMP-8, matrix metalloproteinase-8; PTX3, pentraxin 3.

**Table 2:** **Biological sources, clinical evidence and translational assessment of selected cardiometabolic** **biomarkers.**

Biomarker/Source	Study/Population	Outcome	Effect/Adjustment	Comparators/Added Value	Validation	Clinical Use/AHA Phase/Limits	Refs.
hsCRP; hepatic acute-phase protein; blood.	JUPITER RCT; 17,802 adults without CVD/diabetes; LDL-C < 130 mg/dL, hsCRP ≥ 2 mg/L; median 1.9 years.	First MI, stroke, revascularization, unstable-angina admission or CV death.	Rosuvastatin vs. placebo: HR 0.56 (0.46–0.69); randomized treatment effect, not an hsCRP concentration effect.	hsCRP-based eligibility; no low-hsCRP randomized group. Incremental prediction/calibration: NA.	Multicenter RCT; no prediction-model or test-strategy validation.	Preventive-treatment evidence in a selected population; phases 4–5 benefit of testing unproven. Adiposity/transient inflammation affect hsCRP.	[33]
GlycA; serum acute-phase glycoprotein NMR signal.	METSIM cohort; 8749 Finnish men without diabetes (GlycA: 8716); mean 6.8 years.	Incident diabetes and CVD events.	Reported HR/SD: diabetes 1.37 (1.29–1.46); CVD 1.21 (1.12–1.32). Adjusted *p* < 0.0001/*p* = 0.003; adjusted HRs not tabulated. Covariates: age, BMI, smoking, activity, LDL-C.	hsCRP/IL-1RA compared; IL-6 not measured. Combined-score associations; incremental metrics/calibration: NR.	Single cohort; independent/model validation: NR.	Phase 2 association evidence; complementary prognostic potential. Men only; NMR platform and nonspecific acute-phase signal limit transferability.	[27]
IL-6; immune and nonimmune cells; plasma ELISA.	LURIC cohort; 3300 coronary-angiography referrals; median 9.9 years.	All-cause/CV mortality.	IL-6 >3.2 ng/L with hsCRP ≤ 3.4 mg/L vs. both low: adjusted CV-mortality HR 1.47 (1.10–1.96). Covariates: clinical risks, renal status, albumin, medications, SAA.	hsCRP/SAA comparison. Mortality AUC: IL-6 0.638; three markers 0.635. Clinical-model increment, NRI/IDI, calibration: NR.	Internal/external validation: NR.	Phase 2 association evidence; prognostic potential, phase 3 unestablished. Referral population; assay sensitivity, renal function and adiposity matter.	[136]
Hannum age acceleration; blood DNA methylation, 450K array.	Longitudinal cohort; 179 US veterans, high PTSD prevalence; ~2 years.	Change in latent MetS severity.	Baseline advanced Hannum age: standardized β = 0.17, *p* < 0.001; CI NR. Baseline severity/PTSD considered; DNAm residuals adjusted for age, sex, leukocytes, ancestry.	CRP as parallel trait; no hsCRP/IL-6 incremental comparison. Risk-prediction metrics/calibration: NA.	Independent validation: NR; Horvath-clock associations absent.	Phase 1 with longitudinal phenotype support; biological-aging research. Selected population/cell composition limit extrapolation.	[15]
PBMC miR-155, miR-122, miR-193a; intracellular qPCR.	Cross-sectional; 45 children with obesity and 45 normal-weight controls; 10–18 years.	Obesity, metabolic traits, inflammation.	miR-155–HOMA-IR: unadjusted Spearman r = −0.36, *p* = 0.001; CI/adjusted clinical effect: NR.	hsCRP correlations; IL-6 not assessed. No risk model; incremental metrics/calibration: NA.	Internal/external validation: NR.	Phase 1 cellular evidence, not circulating-RNA diagnosis/prognosis. Puberty, PBMC composition and assay processing require control.	[42]
OxLDL; oxidized LDL; plasma.	Double-blind RCT; red yeast rice–olive extract/placebo; 50 MetS adults (26/24); 8 weeks.	Oxidative/lipid biomarker changes.	Mean OxLDL change ± SEM: −19.35 ± 4.43 vs. +3.65 ± 2.35 U/L; *p* < 0.001. Clinical risk effect: NA.	No hsCRP/IL-6 predictive comparison; incremental metrics/calibration: NA.	Model validation: NA; no outcome validation.	Potential response marker; clinical prediction phase unestablished. No treatment-selection evidence; short follow-up, diet, LDL and assay effects.	[31]
Ceramides/sphingolipids; blood LC–MS/MS.	Uncontrolled Ramadan before–after study; 57 overweight/obese adults; 29–30 days.	Lipid species; inflammatory/metabolic measures.	Ceramides/dihydroceramides unchanged (*p* ≥ 0.11); selected sphingomyelins decreased. Clinical risk effect: NA.	IL-6 as outcome, not comparator. Incremental metrics/calibration: NA.	No prediction-model or outcome validation.	Exploratory response profiling; clinical prediction phase unestablished. No nonfasting control; diet/fasting effects inseparable.	[32]
TMAO; dietary/host–microbial metabolism; plasma.	Multiethnic Cohort cross-sectional analysis; 1653 adults, 60–77 years.	Insulin resistance; lipid, inflammatory and microbiome traits.	Log TMAO–log HOMA-IR: β = 0.08 (SE 0.03), *p* = 0.005. Adjusted for age, sex, ethnicity, activity, body fat, aspirin.	hsCRP as outcome (β = 0.02, *p* = 0.73); IL-6 not assessed. Incremental prediction/calibration: NA.	No prospective model or outcome validation.	Phase 1 metabolic-context evidence, not prognosis. Diet, renal clearance and medication effects; not exclusively microbial.	[16]
LBP-1; host response to bacterial products; blood ELISA.	CLIP secondary analysis; 288 overweight/obese adults, 60–79 years, cardiometabolic disease/mobility limitations; 6 months.	Physical function and inflammation.	Log 400 m walk time per SD LBP-1: coefficient 0.03 (SE 0.01), *p* = 0.001; age/sex/BMI adjusted. Baseline LBP-1 did not predict functional change.	Association after IL-6 adjustment: *p* = 0.003; not incremental prediction. hsCRP comparison: NR; incremental metrics/calibration: NA.	Sensitivity analyses; no independent prediction validation.	Phase 1 host–microbial and functional evidence. Comorbidities and adiposity matter; not a direct permeability test.	[60]
NLRP3-bearing EVs; blood EV fractions/plaque EVs.	26 MetS/23 non-MetS donors; plaques from 12 men; vascular-cell and macrophage experiments.	EV cargo, permeability, migration, proliferation, cytokines.	More NLRP3-positive large EVs in MetS; experimental effects attenuated by MCC950. Clinical risk effect: NA.	IL-6 as experimental response; no hsCRP/IL-6 predictive comparison. Incremental metrics/calibration: NA.	Mechanistic experiments; no prospective outcome validation.	Phase 1 mechanism evidence; pathway research. Isolation, size, source and lipoprotein contamination matter; plaque-progression prediction unproven.	[10]
SII/SIRI; peripheral blood-cell counts.	NHANES mortality cohort; 18,452 adults, CKM 0–4; median 113 months.	All-cause/CV mortality: 2910/941 deaths.	High vs. low, adjusted all-cause HR: SII ≥ 898.21, 1.42 (1.23–1.56); SIRI ≥ 1.23, 1.28 (1.14–1.43). Demographic, socioeconomic/lifestyle, BMI/comorbidity adjustment.	No hsCRP/IL-6 comparison. Five-year AUC: SII 0.579; SIRI 0.662. Clinical-model increment, NRI/IDI, calibration: NR.	1000 bootstraps for cutoff stability; external validation: NR.	Phase 2 association evidence; accessible risk candidates. Sample-derived cutoffs; infection, smoking and blood-cell abnormalities limit specificity.	[12]
MMP-8; neutrophil collagenase; serum ELISA, ng/mL.	Single-center cohort; 127 MetS adults; dietary/behavioral counseling and usual care; 6 months.	Fewer MetS criteria (73/127), not necessarily remission.	Stepwise-model OR 0.970 (0.949–0.993), *p* = 0.009; waist circumference/baseline MetS criteria (4 or 5 vs. 3) adjusted.	No hsCRP/IL-6 comparator. Apparent AUC: MMP-8 0.612 (0.509–0.715); combined model 0.783 (0.701–0.865). Clinical-model ΔC/NRI/IDI: NR. Hosmer–Lemeshow *p* = 0.232; calibration slope/plot: NR.	1000 bootstraps for ORs; corrected AUC: NR; no external cohort.	Phase 2 association evidence; potential follow-up targeting. Pilot cohort, stepwise selection, surrogate outcome; incremental value unproven.	[29]

Entries summarize representative evidence by biomarker; phases apply to the specified population, outcome and use, not all evidence for that marker. AHA phases: 1, proof of concept; 2, prospective validation in independent populations; 3, incremental value; 4, effect on clinical management; 5, improved clinical outcomes; 6, cost-effectiveness. Single-cohort associations contribute to phase 2 without establishing independent validation. The 2026 update also emphasizes calibration, generalizability and implementation [34,153]. NR, not reported in the full text examined; NA, not applicable. Intervals are 95% CIs; SE/SEM are given when CIs were not reported.

## Data Availability

No new data were created or analyzed in this study. Data sharing is not applicable to this article.
